# A Genome-Wide Study of Allele-Specific Expression in Colorectal Cancer

**DOI:** 10.3389/fgene.2018.00570

**Published:** 2018-11-27

**Authors:** Zhi Liu, Xiao Dong, Yixue Li

**Affiliations:** ^1^Department of Epidemiology and Biostatistics, Jiangsu Key Lab of Cancer Biomarkers, Prevention and Treatment, Collaborative Innovation Center for Cancer Personalized Medicine, School of Public Health, Nanjing Medical University, Nanjing, China; ^2^Department of Genetics, Albert Einstein College of Medicine, Bronx, NY, United States; ^3^Key Laboratory of Computational Biology, CAS-MPG Partner Institute for Computational Biology, Shanghai Institute of Nutrition and Health, Shanghai Institutes for Biological Sciences, University of Chinese Academy of Sciences, Chinese Academy of Sciences, Shanghai, China; ^4^Shanghai Center for Bioinformation Technology, Shanghai Industrial Technology Institute, Shanghai, China; ^5^Collaborative Innovation Center for Genetics and Development, Fudan University, Shanghai, China

**Keywords:** allele-specific expression, colorectal cancer, *cis*-regulatory variation, somatic mutation, tumor

## Abstract

Accumulating evidence from small-scale studies has suggested that allele-specific expression (ASE) plays an important role in tumor initiation and progression. However, little is known about genome-wide ASE in tumors. In this study, we conducted a comprehensive analysis of ASE in individuals with colorectal cancer (CRC) on a genome-wide scale. We identified 5.4 thousand genome-wide ASEs of single nucleotide variations (SNVs) from tumor and normal tissues of 59 individuals with CRC. We observed an increased ASE level in tumor samples and the ASEs enriched as hotspots on the genome. Around 63% of the genes located there were previously reported to contain complex regulatory elements, e.g., human leukocyte antigen (HLA), or were implicated in tumor progression. Focussing on the allelic expression of somatic mutations, we found that 37.5% of them exhibited ASE, and genes harboring such somatic mutations, were enriched in important pathways implicated in cancers. In addition, by comparing the expected and observed ASE events in tumor samples, we identified 50 tumor specific ASEs which possibly contributed to the somatic events in the regulatory regions of the genes and significantly enriched known cancer driver genes. By analyzing CRC ASEs from several perspectives, we provided a systematic understanding of how ASE is implicated in both tumor and normal tissues and will be of critical value in guiding ASE studies in cancer.

## Background

Allele-specific expression (ASE) refers to the phenomenon that occurs in diploid or polypoid genomes, where two or more alleles of a gene has an imbalanced expression (Kwaepila et al., 2006; Ge et al., 2009; Heap et al., 2010; Tung et al., 2011). It is common in both humans (Lo et al., 2003) and other organisms (Tung et al., 2011; Graze et al., 2012; Hasin-Brumshtein et al., 2014), and potentially contributes to multiple phenotypes and complex traits (Frazer et al., 2009). Because of the intrinsic power of using two alleles of a gene in the same individual, as controls to reduce the background genetic and environmental effects, ASE is also an accurate and sensitive marker for *cis*-regulatory variation (Pastinen, 2010). For example, an ASE can indicate a heterozygous variant within the translated region, resulting in a modified or truncated protein (Kukurba et al., 2014); at a regulatory site, it can cause differential binding of transcription factors or epigenetic modifiers (Prendergast et al., 2012; Reddy et al., 2012); or at a splice site or UTR, it can affect transcript processing (Li et al., 2012).

Allele-specific expressions are also frequently observed in tumors (Valle et al., 2008; Curia et al., 2012; Walker et al., 2012; Wei et al., 2013). ASE was first proposed as a direct approach for connecting a genotype to disease susceptibility in 2002 (Yan et al., 2002). In 2013 it was discovered that ASE, at the death-associated protein kinase 1 (*DAPK1*) gene locus, was potentially predisposed to chronic lymphocytic leukemia (CLL) using a single-nucleotide primer extension (SNuPE) and MALDI-TOF mass spectrometry (Wei et al., 2013). In colorectal cancer (CRC), a decrease in expression of one adenomatous polyposis coli tumor suppressor (*APC*) gene allele, leads to the development of familial adenomatous polyposis (Curia et al., 2012). In addition to *APC*, ASE of transforming growth factor beta receptor 1 (*TGFBR1*), which leads to reduced expression of the gene, can also cause an increased risk of CRC (Valle et al., 2008). In addition to the association with cancer risk, ASE also affects the prognosis and outcome of cancer patients. For example, the monoallelic expression of TP53 and IDH1 was found to determine the oncogenic progression and survival in brain tumors (Walker et al., 2012).

With the development of large-scale transcriptome sequencing, the systematic analysis of the ASE in the transcriptome was achieved at the single nucleotide resolution (Tuch et al., 2010; Smith et al., 2013). To date, several studies have reported genome-wide ASE, in human, mice and cell lines, and identified hundreds of genes exhibiting ASE (Heap et al., 2010; Smith et al., 2013; Hasin-Brumshtein et al., 2014). However, little is known about genome-wide patterns of ASE in tumor tissues. In this study, we carried out an ASE study in a cohort of 59 patients with CRC (Seshagiri et al., 2012) and revealed the comprehensive landscape of ASE in CRC patients.

## Materials and Methods

### Data Preprocessing

RNA and Exon sequencing data of matched human colorectal tumor-normal samples was downloaded from the European Genome-Phenome Archive (EGA) under accession number EGAS00001000288 (Seshagiri et al., 2012). Fifty-nine pairs of samples correctly processed were retained for ASE analysis.

Quality controlled DNA and RNA sequencing data was mapped with bowtie2 (Langmead and Salzberg, 2012) with default parameters to report the best alignment. The base qualities were then recalibrated using the procedure recommended by GATK (DePristo et al., 2011).

Somatic mutations were called with both Mutect (Cibulskis et al., 2013) and Varscan (Koboldt et al., 2009), and the intersection was considered a reliable result and used for the following analysis. Germline SNVs were called using the GATK best practices from DNA sequencing data, and filtered using the flowing four criteria to obtain a final SNV list ready for ASE analysis.

(1) SNVs cluster together;
(2) SNVs covered by less than 20 reads;
(3) SNVs located within repeated regions;
(4) SNVs located within non-coding regions;
(5) SNVs were identified as a somatic mutation in exon sequencing data.

Allele counts for each germline SNV and the somatic mutation in DNA and RNA sequencing data, were generated with SAMtools (Li et al., 2009) in a pileup format.

The list of germline SNV and somatic mutation, as well as the corresponding pileup files were subjected to cisASE for ASE identification, respectively.

### ASE Identification

Allele-specific expression SNVs and genes were identified by the cisASE pipeline (Liu et al., 2016). SNVs with a coverage of less than 10 in RNA or DNA sequencing data were filtered. SNVs or genes with a log likelihood ratio (LLR) value more than the LLR cutoff, at a significance level of 0.01, were defined as ASE. In addition, genes with a heterogeneity *p*-value less than 0.05, which indicates inconsistent ASE status of SNVs within the gene, were excluded from further analysis.

### Identifying ASE Hotspots

Allele-specific expression counts and frequency was calculated in consecutive sliding windows with fixed sizes along the genome. We randomly assigned ASE labels to the SNVs across chromosomes, according to the total ASE frequency. By repeating this process 1000 times, we obtained a null distribution of ASE density in each of the sliding windows. A *p*-value was derived by counting the number of times the number of ASEs in the window after perturbation, exceeded the observed ASE, and adjusted it with an add-one smoothing. These *p*-values were then corrected for multiple tests using the Benjamini-Hochberg method.

### Group of ASE Somatic Mutation

We mapped the ASE somatic mutations to genes and then classified the genes into two categories, i.e., genes with over-expressed mutant alleles and genes with under-expressed mutant alleles. Genes harboring multiple somatic mutations with conflicting mutant allele expression, were excluded from the following analysis. Gene expression profiles were generated with tophat2 (Kim D. et al., 2013) and cufflinks (Trapnell et al., 2012) software. For genes in each group, we compared the FPKM value of both tumor and normal tissues of patients with the somatic mutation, and defined the FPKM fold change of 2 and 1/2 as the threshold of up-regulated and down-regulated expression in tumor samples. This resulted in three groups for each category according to the gene expression.

### Identifying Somatic ASE Genes

We counted the number of ASE somatic events (s_i_) and the number of total tested pairs (t_i_) in a population of 118 individuals, for each gene. We refer to the ASE somatic event (s_i_) as the gene showing ASE in a tumor sample but not in the matched normal sample. In addition, we refer to the tested pairs (t_i_) as the sample pairs, and the gene is tested in both matched tumor-normal samples. The expected ASE somatic event rate was calculated by the following equation,

$$f=\sum_{i=1}^{n}S_{\text{i}}/\sum_{i=1}^{n}t_{\text{i}},$$

where *n* is the number of genes.

The expected number of the ASE somatic event for each gene, was calculated as the product of the total tested pairs and the expected ASE somatic event rate (f^∗^t_i_). A *p*-value was obtained for each gene using the Poisson distribution and the observed and expected number of ASE somatic events (*P*[X ≥ x]). These *p*-values were corrected for multiple testing using the Benjamini-Hochberg method and genes that had a corrected *p*-value <0.05 were called a somatic ASE gene.

## Results

### Increased ASE Level in Tumor Samples

**FIGURE 1:**
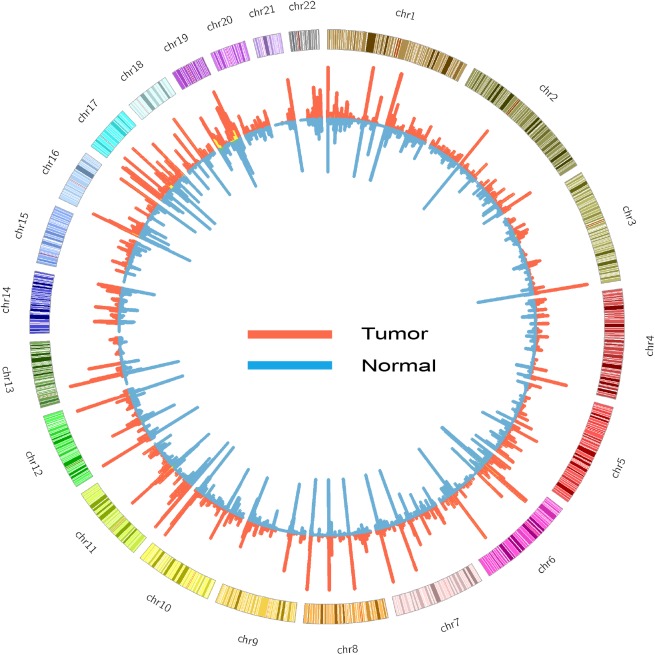
Circos diagram of ASE detected in tumor (red) and normal (blue) samples. The height of histogram indicates the counts of ASE in a window of 1 million base pair.

We identified SNV and gene level ASEs in both normal and tumor tissues of 59 CRC patients with our previously developed pipeline for ASE identification (Kukurba et al., 2014; Figure 1 and Supplementary Table S1). The major steps included sequence alignment, variant calling, ASE detection using cisASE (Kukurba et al., 2014), and further filtration (see section “Materials and Methods” for details). We detected 431 (*SD* = 133.3) SNV-level ASEs per normal tissue and 477 (*SD* = 181.6) per tumor tissue, and 137 (*SD* = 39.3) and 216 (*SD* = 108.5) gene-level ASEs per normal and tumor tissue, respectively (Supplementary Table S1). The frequency of ASE SNVs (a ratio of number of ASE SNVs to number of non-ASE SNVs) in normal tissue is in agreement with previous studies (Zhang et al., 2009; Skelly et al., 2011).

**FIGURE 2:**
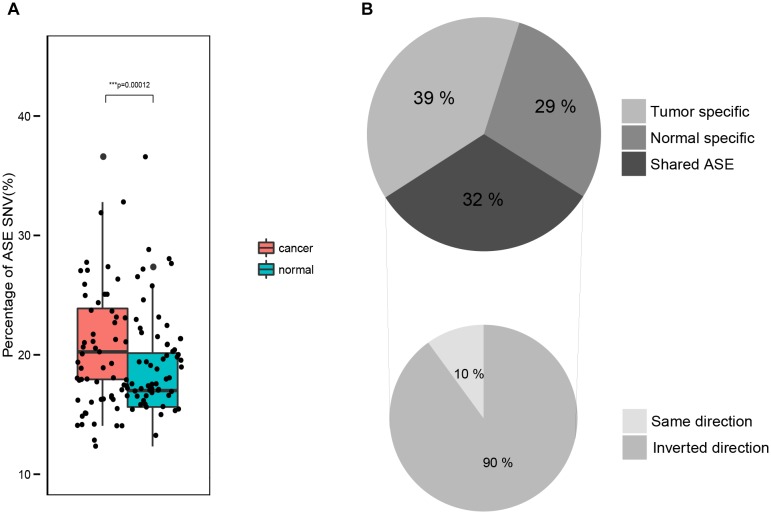
Comparison of ASE in normal and tumor tissues. **(A)** The fraction of ASE in normal and tumor samples. **(B)** Intersection of ASE identified in paired normal and tumor samples.

We compared the portion of sites exhibiting an ASE in tumors with its matched normal tissues. On average, 20.0% of the SNVs in tumor samples and 16.8% in normal samples exhibited an ASE (two-tailed paired *t*-test, *p*-value = 7.1e–09), indicating a significantly higher ASE level in tumor samples than in normal samples. When only testing the SNVs identified in tumor and normal tissues, the results were the same, i.e., a significantly higher portion of the ASE in tumor samples (21.6%) than in the normal samples (18.1%; two-tailed paired *t*-test, *p*-value = 1.2e–04; Figure 2A).

For each tumor-normal pair, we found that 68% of the ASEs are either normal (29%) or tumor (39%) specific (Figure 2B). And the remaining 32% of the ASEs are shared by both the tumor and normal samples (Figure 2B), most of which had the same ASE direction. This indicates that the majority of ASEs (about 2/3) are dynamic in tumorigenesis while the other 1/3 ASEs are consistent.

**FIGURE 3:**
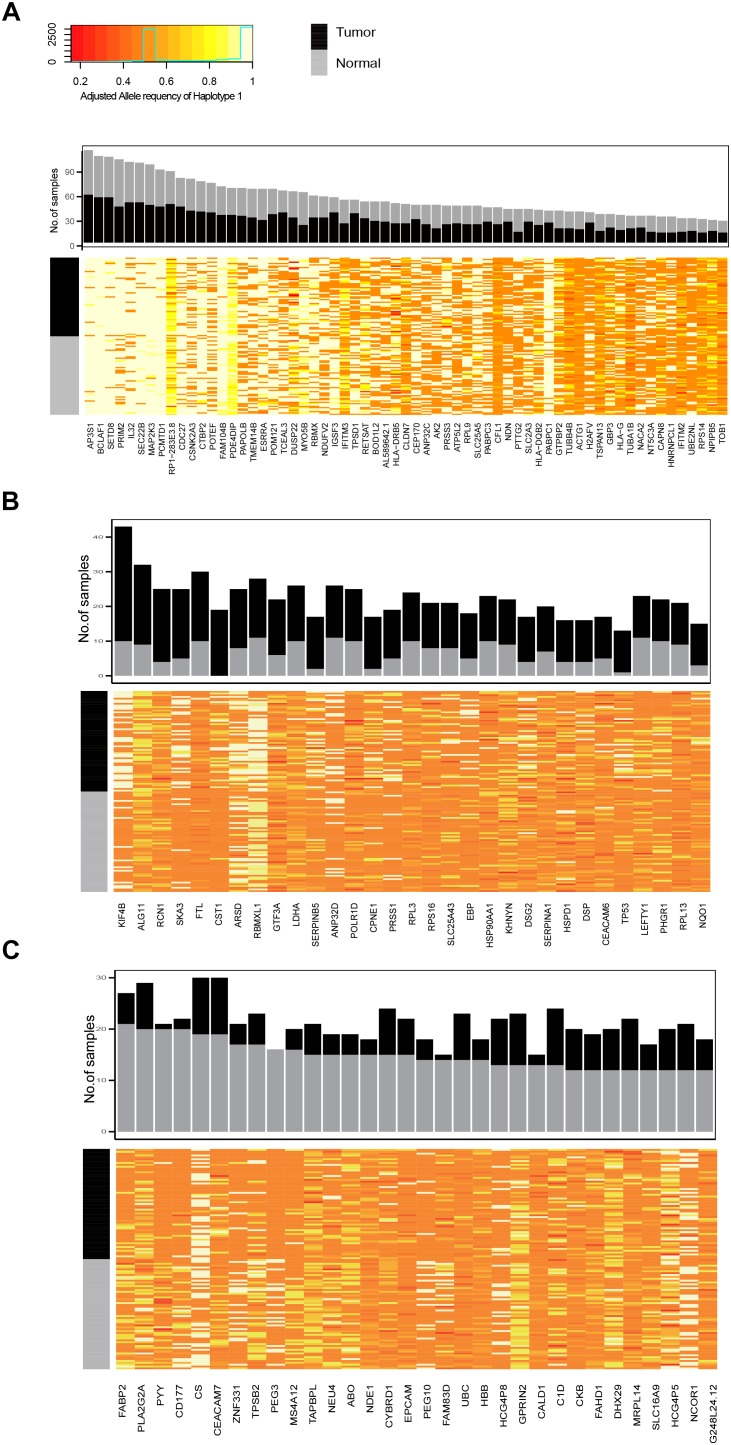
The frequency of recurrently observed ASE genes and heatmap of their allele ratio among samples. **(A)** Genes shared by tumor and normal samples, **(B)** ASE genes recurrently observed in tumor samples; **(C)** ASE genes recurrently observed in normal samples.

Next, we identified genes with recurrent ASE events in tumor and normal samples. There were 94 and 95 genes with ASE events in at least 20% of the tumor and normal samples, respectively, of which 63 genes were shared by both tumor and normal samples (common ASE genes) (Supplementary Table S2 and Figure 3). The allele ratio of recurrent of ASE genes was significantly segregated from the background and the total pool of ASE genes (Supplementary Figure S1) and the average major haplotype allele ratio of common ASE genes was 0.92.

The ASE genes that was mostly recurrently observed in both tumor and normal samples had a high allele imbalance, such as AP3P1, BCLAF1, STED8, PRIM2, IL32, SEC22, and MAP2K3 (Figure 3A). The recurrent ASE genes in tumor samples include Chromosome-Associated Kinesin KIF4B, spindle and kinetochore associated complex subunit 3 (SKA3) and so on. We also found that the ASE of TP53 was specifically and recurrently observed in tumor samples (observed in 12 tumor samples and 1 normal sample). There were 32 recurrent ASE genes observed in normal samples. For example, PYY, CD177, PEG3, and FAM83D, were observed in more than 11 normal samples, while less than 3 were observed in tumor samples.

### The ASE Hotspots in the Normal and Tumor Genome

Variants on the *cis*-regulatory element on the genome, tend to affect the expression of one or more genes nearby (Pastinen, 2010), and if the variation is heterozygous, the genes regulated by it will exhibit an ASE, therefore we prioritized the existence of such variations by identifying clusters of the ASEs on genomic regions. We calculated the ASE density and frequency in the tumor and normal samples, by using a sliding window approach with a window size of 100k base pair (bp) and a step size of 10 kbp. Windows with an adjusted *p*-value <0.05 were kept, and overlapping windows were manually checked and merged, to get focal hotspot regions.

**FIGURE 4:**
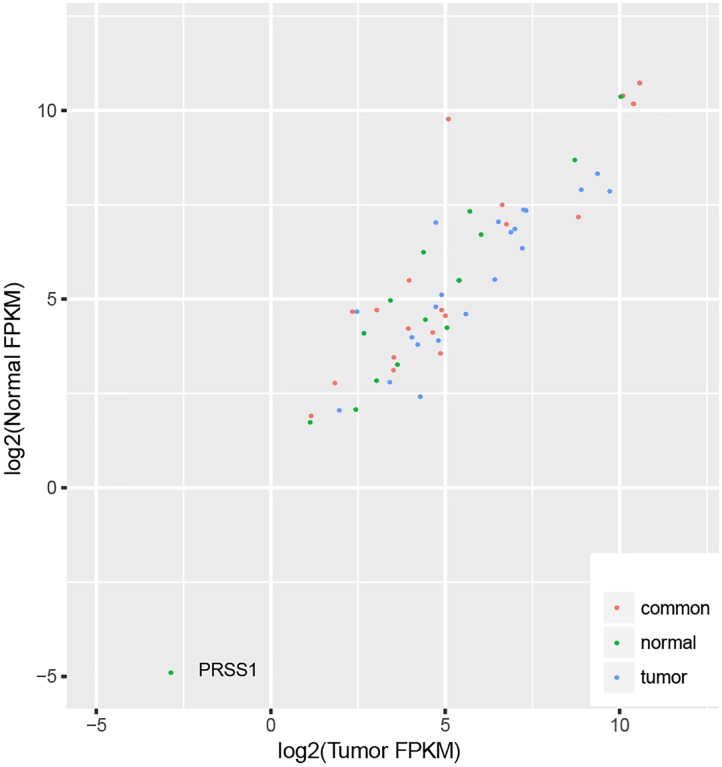
Gene expression in tumor and normal samples for genes located in ASE hotspots.

We identified 32 ASE hotspots in normal samples (Supplementary Table S3) and 27 in tumor samples (Supplementary Table S4), affecting a total of 57 genes (Supplementary Figure S2), which resulted in 4.0% (723 out of 17866) and 4.4% (748 out of 17866) of the ASE SNVs identified in normal and tumor samples, respectively. There were 21 genes located within hotspots identified in both normal and tumor samples, as well as 22 and 14 genes located within the hotspots specific to tumor and normal samples, since the tumor or normal differential expression might result in a different power of ASE detection. We checked the expression of all these genes in tumor and normal samples (Supplementary Table S5), and found no difference of the tumor and normal FPKM ratio among the three groups of genes (Kruskal–Wallis test *p*-value = 0.07), indicating the difference of the ASE hotspots in tumor and normal samples did not result from the different detection powers, due to the expression difference. In addition, one gene with relatively low expression (PRSS1, FPKM < 0.1) was excluded (Figure 4).

To investigate the biological process affected by the ASE, we conducted the GO and KEGG enrichment analyses for the ASE genes. The ASE genes shared by tumor and normal tissues were significantly enriched in antigen processing. The significantly enriched GO and KEGG terms of tumor specific ASE genes were closely associated with immune activity. However, the normal ASE genes were not enriched in specific functions. The results impose the possibility that the ASE plays a role in maintaining regular immune activities, and an excessive ASE event was activated in tumor tissues.

Among the genes shared in normal and tumor tissues, several genes involved in polymorphism, or which were reported to be related to cancer predisposition or progression were included, such as, the human leukocyte antigen (HLA) on chromosome 6, members of the mucin gene family (MUC) on chromosome 3, and the MAP2K3 and CDC27 locus, which is involved in the cell proliferation and cell cycle on chromosome 7. Three members of the carcinoembryonic antigen (CEA) family (CEACAM5, CEACAM6, and CEACAM7) were also included.

**FIGURE 5:**
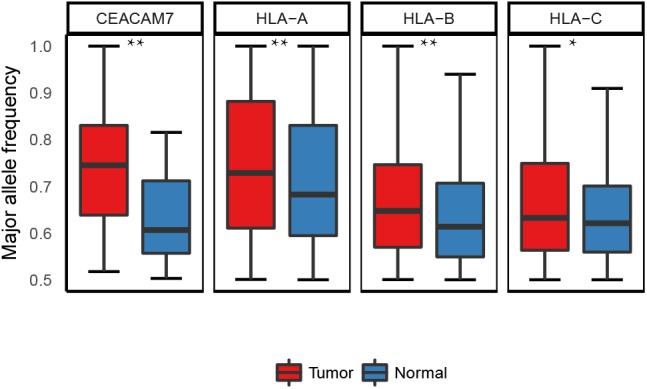
Allele ratio of the four shared ASE genes which exhibited differential allele ratio in tumor and normal samples. ^∗^*p* < 0.05, ^∗∗^*p* < 0.01.

Though 35.6% (21 out of 59) of the ASE genes were common in both normal and cancer tissues, it was reported that the change in the allele ratio of the ASE can also lead to phenotypic diversity, for example, a study reported that the proportion of the JAK2 V617F mutant allele in RNA levels is significantly associated with distinct subtypes of BCR/ABL-negative myeloproliferative neoplasms (MPNs) (Kim H.R. et al., 2013). Therefore, we tested whether there are similar cases in ASE genes between the tumor and normal tissues. We found that four out of the 21 shared ASE genes (HLA-A, HLA-B, HLA-C, and CEACAM7) and showed significant differences in the allele ratios between tumor and normal tissues (paired *t*-test; Supplementary Table S6). Three of the four genes belong to the HLA family, i.e., HLA-A and HLA-B, and HLA-C, and all revealed a lower allelic heterozygosity in tumor tissues (Figure 5). Loss of heterozygosity (LOH) of the HLA loci was reported in many cancers (Maleno et al., 2002; Wang et al., 2006; Zollikofer et al., 2014). In our case, these loci are heterozygous at the DNA level, while at the mRNA level, one of the copies showed a significantly reduced expression compared to the other one. The results suggest the possibility that in tumor tissues, the allele-specific regulation on the transcriptional level may lead to a similar consequence as the LOH.

The other 18 shared ASE genes, showed no difference in the allele ratio, between normal and tumor tissues (Supplementary Figure S3), indicating that most of the shared ASEs are conserved during tumorigenesis. However, because the normal tissues we studied were obtained from CRC patients, ASE genes shared by tumor and normal tissues can be involved either in normal physiological functions or associated with tumor predisposition. Since it is hard to obtain gut tissue samples from healthy people, we cannot distinguish these two possibilities.

Of the twenty-two genes located in the tumor-specific hotspots (Supplementary Table S4), several were reported to play an important role in tumor progression. For example, over-expression of the FAT1 was observed in different tumors including in DCIS breast cancer (Kwaepila et al., 2006), melanoma (Sadeqzadeh et al., 2011) and leukemia (de Bock et al., 2012). MKI67 is a protein that is widely used as a marker for cell proliferation, and its increased expression in human cancer specimens generally denotes an aggressive phenotype (Cidado et al., 2016). The observed allele specific expression of these phenotypes may help to prioritize the underlying mechanisms which contribute to the abnormal expression in tumors. Furthermore, 14 genes (ACSF3, AHNAK, APOBR, CCBL2, CLN3, EPPK1, FAM104B, FUT2, HLA-DRA, HLA-G, MUC12, NBPF1, RASIP1, RBMXL1, and SLC25A5) were located in the normal-specific hotspots (Supplementary Table S3), which suggests that precise control of the ASE may be important for maintaining the normal function of cells. These results might provide opportunities for mining new therapy targets.

### Overexpressed Allele With Somatic Mutations in Tumors

Somatic mutations (missense mutations and non-sense mutations) within the coding region may lead to abnormal protein products. However, the impact of a heterozygous coding SNV depends on whether the SNV-containing allele is transcribed to the RNA. In addition, clinical therapy-selection for targeted drugs, often assay mutations using DNA as an analyte, such as KRAS assays designed to identify responders to anti-EGFR therapy (Allegra et al., 2009). However, if the mutant allele is selectively lost in the transcript, the mutation may not have a therapeutic impact and the merit of using a DNA-based assay for clinical decision-making may be problematic. The above are the major reasons for us to further analyze the allelic expression of somatic mutations in tumors. A genome-wide study in mouse tumor cell lines reported that mutations are transcribed in proportion to their DNA allele frequency (Castle et al., 2014). However, to our knowledge, a genome-wide study of the relationship between DNA and RNA mutation allele frequency in tumor tissues, has not been done.

**FIGURE 6:**
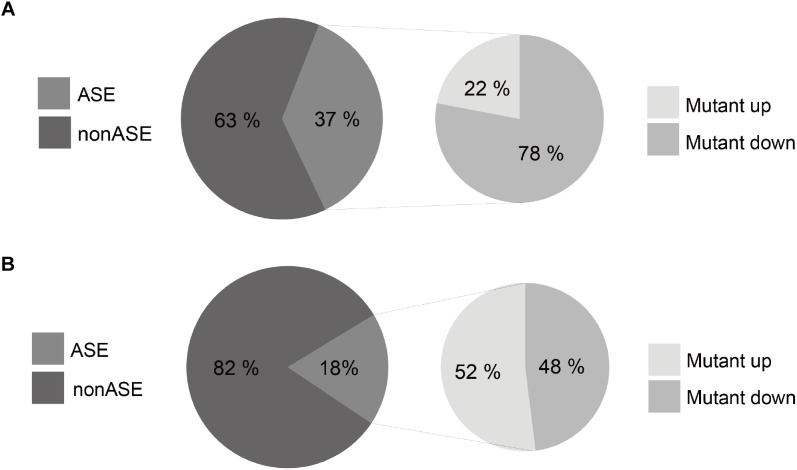
Statistics of ASE for somatic mutations **(A)** and germline polymorphisms **(B)**.

We found that 37.5% of the 2,754 somatic mutations exhibit an ASE in the colorectal tissues (Figure 6A), which is more than two times higher than that for germline polymorphisms (18%) (Figure 6B). This indicates a significant imbalance of the allelic expression for somatic mutations. Furthermore, 78% of the ASE somatic mutations over-expressed mutant alleles, comparing to a proportion of 52% for germline polymorphisms (chi-square test *p*-value <2.2e-16). The results reveal that gene copies with somatic mutations have prevailing expression superiority compared to the wild type ones in tumor tissues.

**FIGURE 7:**
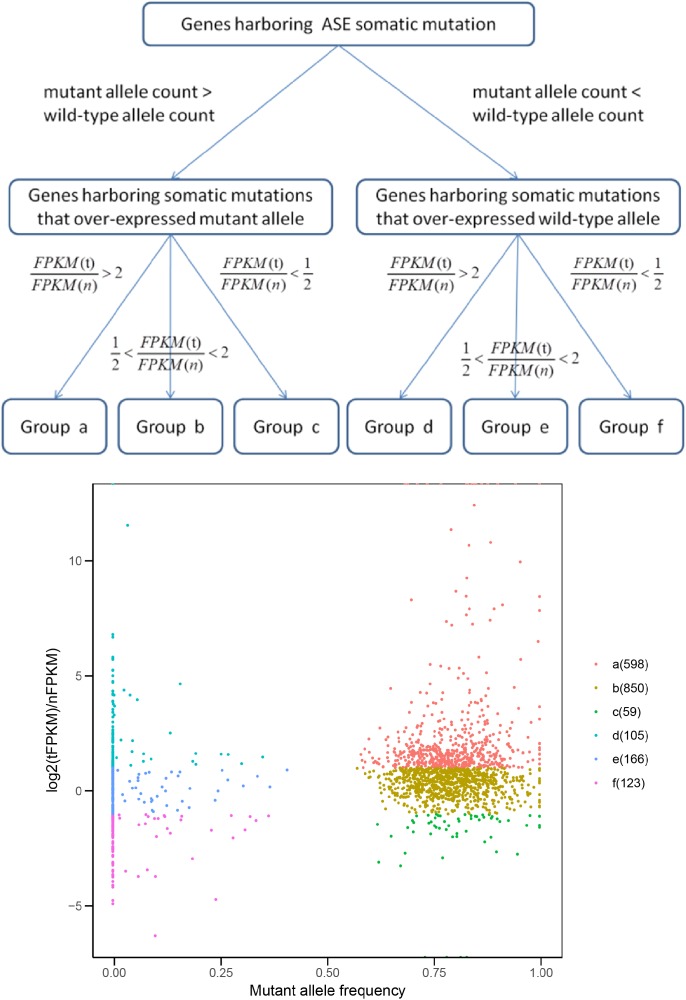
Groups of genes harboring ASE somatic mutations. FPKM (t) and FPKM (n) represent the FPKM value of gene in tumor and its matched normal tissue, respectively.

Next, we explored the functional significance of the ASE somatic mutations with a different mutant/wild-type allele expression pattern. We mapped the ASEs to genes and classified them into six groups according to the alteration of both mutant allele expression and total gene expression in tumor tissues (Figure 7 and Supplementary Table S7; Materials and Methods).

Ideally, if an ASE somatic mutation is functional, the direction of the mutant allele expression change should be the same as the direction of the gene expression change in tumor cells, compared with normal cells (Group a and f in Figure 7). Genes which exhibited the ASE somatic mutation but an unchanged total gene expression (Group b and e in Figure 7) might be regulated by other trans-regulatory factors, and the effects of the ASE were buffered. Those conflicting with the somatic allele expression and tumor gene expression (Group c and d in Figure 7) were possible artificial results, or the ASE was a random event without functional significance.

As expected, only the genes in Groups a were farely significantly enriched in KEGG pathways (Du et al., 2014; Table 1). Group a, which contain genes over-expressing mutant allele and showing an up-regulated gene expression level in tumor samples compared with the matched normal sample, is enriched in the DNA replication and mismatch repair pathways. Dysfunction of the DNA replication and DNA mismatch repair pathways are implicated in many cancer types (Boyer et al., 2016; Puigvert et al., 2016), which initiates cancer or promotes cancer cell proliferation (Padmanabhan et al., 2004; Dudderidge et al., 2007). The average mutant allele fraction for the genes enriched in these two pathways is 80%, indicating a widely over-expressed mutant allele. This suggests that in tumor tissues, genes involved in the DNA replication and DNA mismatch repair pathways, tend to selectively express mutant proteins with abnormal functions, which may compete with normal proteins to disrupt normal signal pathways, or decrease the dosage of normal proteins for normal functions.

**Table 1:** Enriched KEGG pathways for genes in group a and f.

Type a			
Term	ID	Adj.pvalue	Genes
DNA replication	hsa03030	0.019631059	RFC3, MCM7, RFC1, POLD2, MCM3, RNASEH2A
Mismatch repair	hsa03430	0.026586385	EXO1, RFC3, RFC1, POLD2, MLH3

**Type f**			

Term	ID	Adj.pvalue	Genes
Focal adhesion	hsa04510	0.017378214	TLN1, TNC, COL6A3, ZYX, THBS1, FLNA, MYLK

Genes in Group f, which contain genes with under-expressed mutant alleles and down-regulated gene expression in tumor samples, compared with matched normal ones, are enriched in the focal adhesion signal pathway. The genes enriched in the focal adhesion pathway showed limited mutant allele fractions only 10% of the two alleles, suggesting that mutation-containing alleles are effectively silenced by epigenetic and chromatin modification mechanisms (Jaenisch and Bird, 2003) or mutation-containing transcripts are degraded by activating RNA surveillance mechanisms (Rehwinkel et al., 2006), resulting in an overall decrease of gene expression levels and thus an abnormal signal pathway.

### Somatic ASE Genes Are Enriched in Known Cancer-Related Genes

Genes specifically exhibiting the ASE in cancer tissues are likely linked to somatic variations in regulatory regions. In order to detect genes with an excess of somatic *cis*-regulatory events, we used matched tumor and normal samples to identify genes specifically and significantly exhibiting ASE in tumor samples (which we defined as the “somatic ASE gene”). We found 50 somatic ASE genes (Supplementary Table S8), which significantly enriched TCGA pan-cancer drivers (Gonzalez-Perez et al., 2013) (five pan-cancer drivers *p*-value = 0.010) and CRC drivers (Gonzalez-Perez et al., 2013) (two CRC drivers *p*-value = 0.04), indicating that the tumor specific ASE genes analysis catches known cancer genes, and has the potential to be a complementary method for driver detection. Next we compared the somatic ASE gene with differential expressed genes (DEG) between tumor and normal samples (Supplementary Table S9), and found that they significantly enriched in DEGs (fisher exact test *p*-value = 5.0e–07, odds ratio = 3.22).

## Discussion

The ASE is a measure of the effect of genetic variants on gene expression, that does not require any assumption on the genetic structure of the population studied, and hence a direct measurement of how gene-expression changes at the individual level (Yan et al., 2002). The development of next generation sequencing technologies and our unbiased computation method cisASE (Kukurba et al., 2014) have enabled us to characterize this genome-wide landscape of the ASE in tumor and normal tissues of CRC patients from diverse perspectives.

The higher incidence of the ASE in tumor samples than that of normal samples is consistent with the fact that gene expression in tumor cells is under more complicated *cis*-regulation (Maurano et al., 2012). Furthermore, 29 and 39% of the ASE SNVs were specific to either normal or cancer samples, respectively, indicating both the gain and loss of *cis*-regulatory variation as possible contributors to tumor initiation or development. We also observed a high percentage (32%) of ASEs shared by normal and tumors tissues of patients, which might be a mixture of CRC preposition sites, as well as sites where ASE play a role in maintaining regular cellular function. Since it is difficult to obtain gut tissue samples from healthy people to distinguish these two categories of ASE, some researchers suggest using blood samples from normal healthy people (Valle et al., 2008). However, *cis*-regulatory variation is a tissue dependent feature, so is the ASE (GTEx Consortium, 2015), therefore, using a different tissue as control might result in high false discovery rates. Creative and accurate methods are needed to further explore cancer risk sites from regular sites.

By summarizing the ASE in a region-based fashion, we identified the ASE hotspots under true and recurrent *cis*-regulation in the studies samples. Although the majority of the ASE hotspots, including the HLA loci, were shared by both normal and tumor tissues, four of the HLA genes revealed a significant lower heterozygosity in the tumor tissues compared with normal tissues. The LOH in the short arm of chromosome 6 is the most frequent mechanism contributing to the HLA haplotype loss in human cancer, which is a tumor escape mechanisms from the host’s immune surveillance system (So et al., 2005). The selective expression of one allele of the HLA gene might be another mechanism that contributes to the HLA haplotype loss in cancer.

One category of targeted drugs, is the targeting of specific genes with or without certain somatic mutations, such as osimertinib targeting at EGFR (with EGFR T790M mutation) and afatinib targeting at EGFR(with EGFRL858R mutations) in non-small cell lung cancer, vemurafenib targeting at BRCA(with BRAF V600 mutation) in melanoma, and panitumumab targeting at EGFR (with KRAS will type) in CRC. A DNA assay is usually used to test whether a specific gene mutation codes the target. However, an RNA level expression is not necessary a faithful replication of the DNA. We found that 38% of the somatic ASE exhibited the ASE, indicating that the DNA-assay based therapy-selection might be problematic. Somatic mutations and mutant allele that followed the same direction as the total gene expression, i.e., Group a and f, were enriched in important signal pathways involved in tumor initiation and progression. However, mutations belonging to other groups may also have biological implications, are not significantly enriched in the KEGG pathways, since we cannot exclude the possibility that, in some cases, homeostatic or feedback mechanisms act to constrain the total expression so that an imbalance in allelic expression does not change the total output.

Somatic ASE genes were regulated by *cis*-regulatory elements with somatic variations, which may be the driver mutation implicated in cancers, the fact that the identified somatic ASE genes enriched pan-cancer and CRC driver genes, support this speculation.

In this study, we focused on the ASE of protein coding regions. However, in recent years, lncRNAs were reported to be involved in gene regulation and other cellular processes (Quinn and Chang, 2016). With an ASE analysis, Almlof et al. (2014) found that 22.9% (258 out of 1122) of intergenic lncRNAs were regulated by *cis*-rSNP in human primary monocytes, which is comparable to our analysis. Though the number of lncRNAs exceeded the protein coding genes, because of a much lower expression (Iyer et al., 2015), a higher sequencing depth and more sensitive detector is required to quantify ASE in lncRNAs more efficiently.

## Conclusion

By applying the ASE studies in CRC patients, we found a higher incidence of the ASE in tumor tissues, which implicated more complicated *cis*-regulation in tumors. ASEs under recurrent *cis*-regulation were enriched as hotspots on the genome and the majority of the genes (∼63%) involved in the hotspots, were previously reported to have complex regulatory elements, or were implicated in tumor progression. In addition, the ASE analysis of somatic mutation revealed a significant increased ASE rate for somatic mutations, and genes harboring such somatic mutations were enriched in important pathways implicated in CRC (DNA replication and focal adhesion). Furthermore, the somatic ASE genes analysis catches known cancer genes.

In summary, this study provides a systematic understanding of how the ASE is implicated in tumors and a schema of the application of the ASE studies in patients with cancerous tumors.

## Data Availability

The datasets supporting the conclusions of this article are included within the article and its additional files. Raw RNA and Exon sequencing data were downloaded from the European Genome-Phenome Archive (EGA) under accession number EGAS00001000288 by proper application.

## Author Contributions

ZL and XD conceived the study and wrote the manuscript. ZL carried out all the analysis in this study. YL supervised the study and revised the manuscript. All authors read and approved the final manuscript.

## Conflict of Interest Statement

The authors declare that the research was conducted in the absence of any commercial or financial relationships that could be construed as a potential conflict of interest.

## Supplementary Material

FIGURE S1 The allele ratio of recurrent of ASE genes was significantly segregated from the background and the total pool of ASE genes.

FIGURE S2 ASE frequency across chromosomes.

FIGURE S3 The comparison of allele ration of 18 shared ASE genes between normal and tumor tissues.

TABLE S1 SNV and gene levels of ASE in the normal and tumor tissues.

TABLE S2 Genes with recurrent ASE events in tumor and normal samples.

TABLE S3 ASE Hotspots in normal.

TABLE S4 ASE Hotspots in Cancer.

TABLE S5 Expression for genes in hotspots region.

TABLE S6 Differences in allele ratios between tumor and normal tissues.

TABLE S7 Allele ratio and FPKM for somatic ASE and involved genes.

TABLE S8 Somatic ASE genes.

TABLE S9 Gene expression of somatic ASE genes in tumor and normal samples.
