# Supplementary figures and images for: A Genome-Wide Study of Allele-Specific Expression in Colorectal Cancer
Source: Front Genet. 2018 Nov 27;9:570. doi: 10.3389/fgene.2018.00570 (PMC6277598; doi:10.3389/fgene.2018.00570)

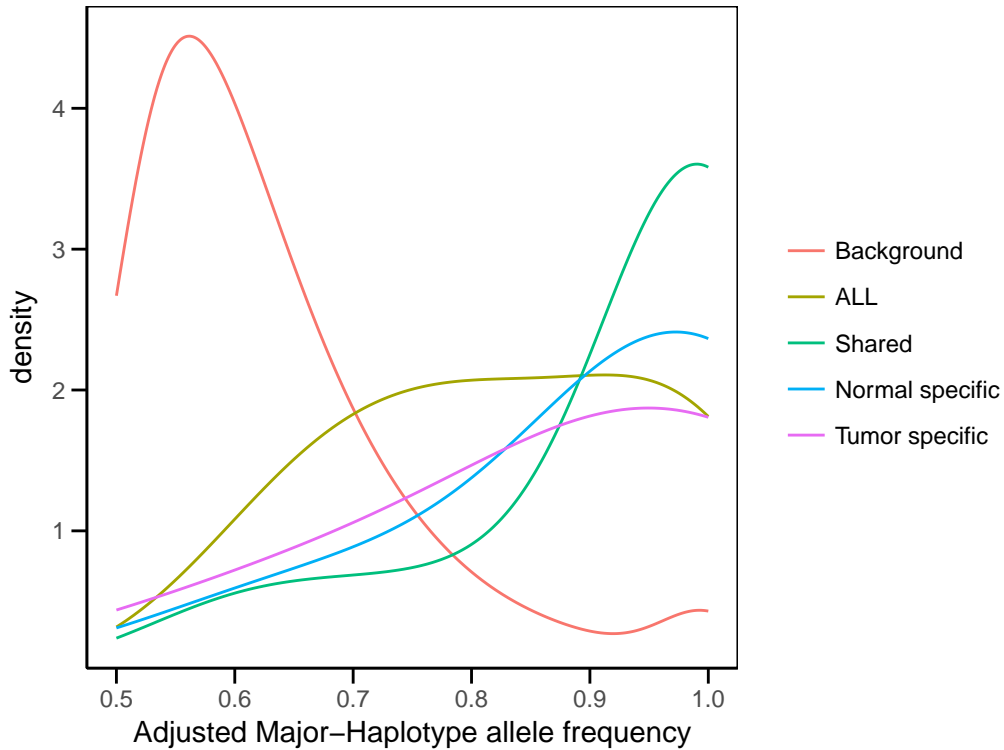

Supplement: FIGURE S1 — The allele ratio of recurrent of ASE genes was significantly segregated from the background and the total pool of ASE genes. [file Data_Sheet_1.PDF]

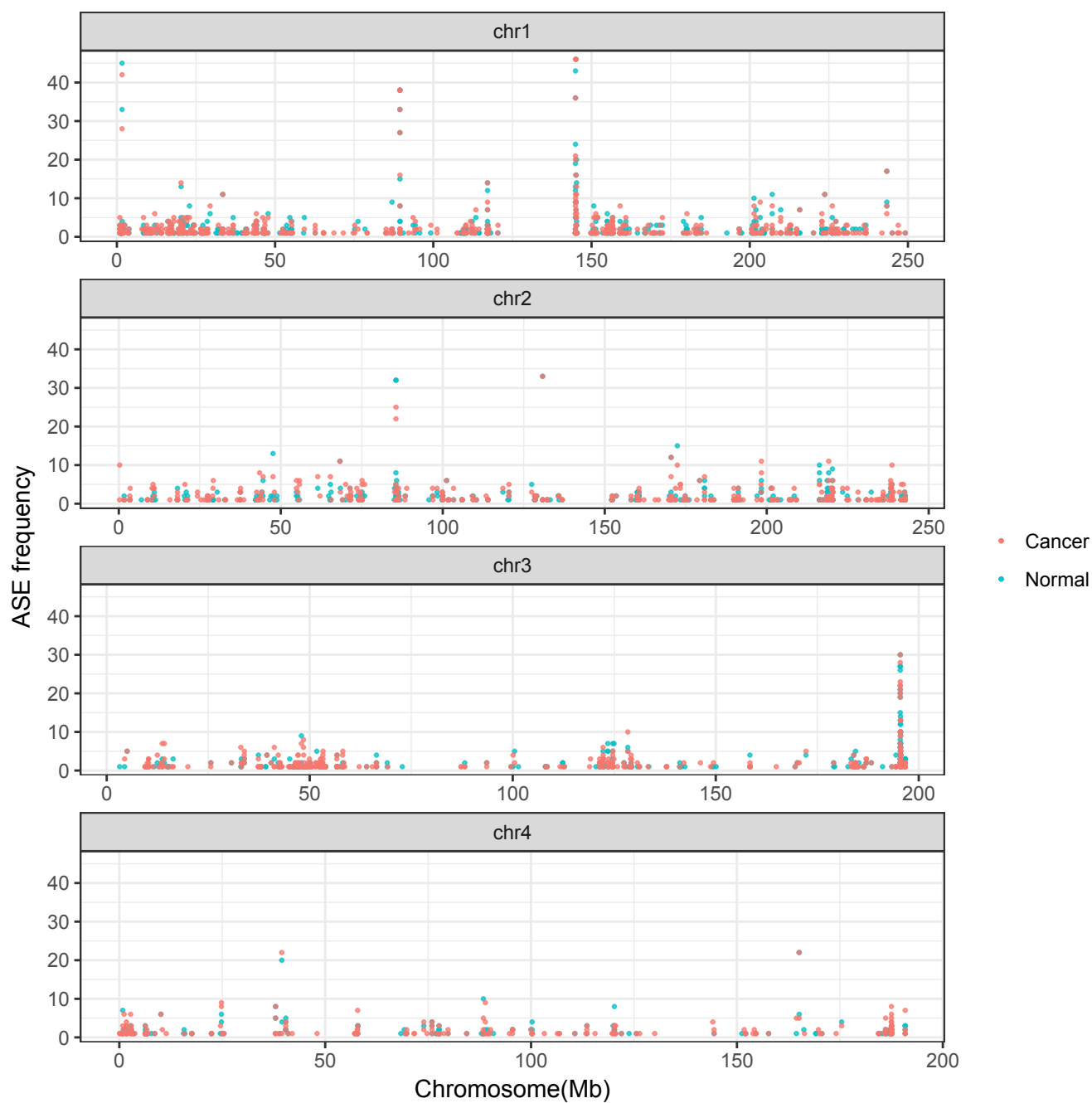

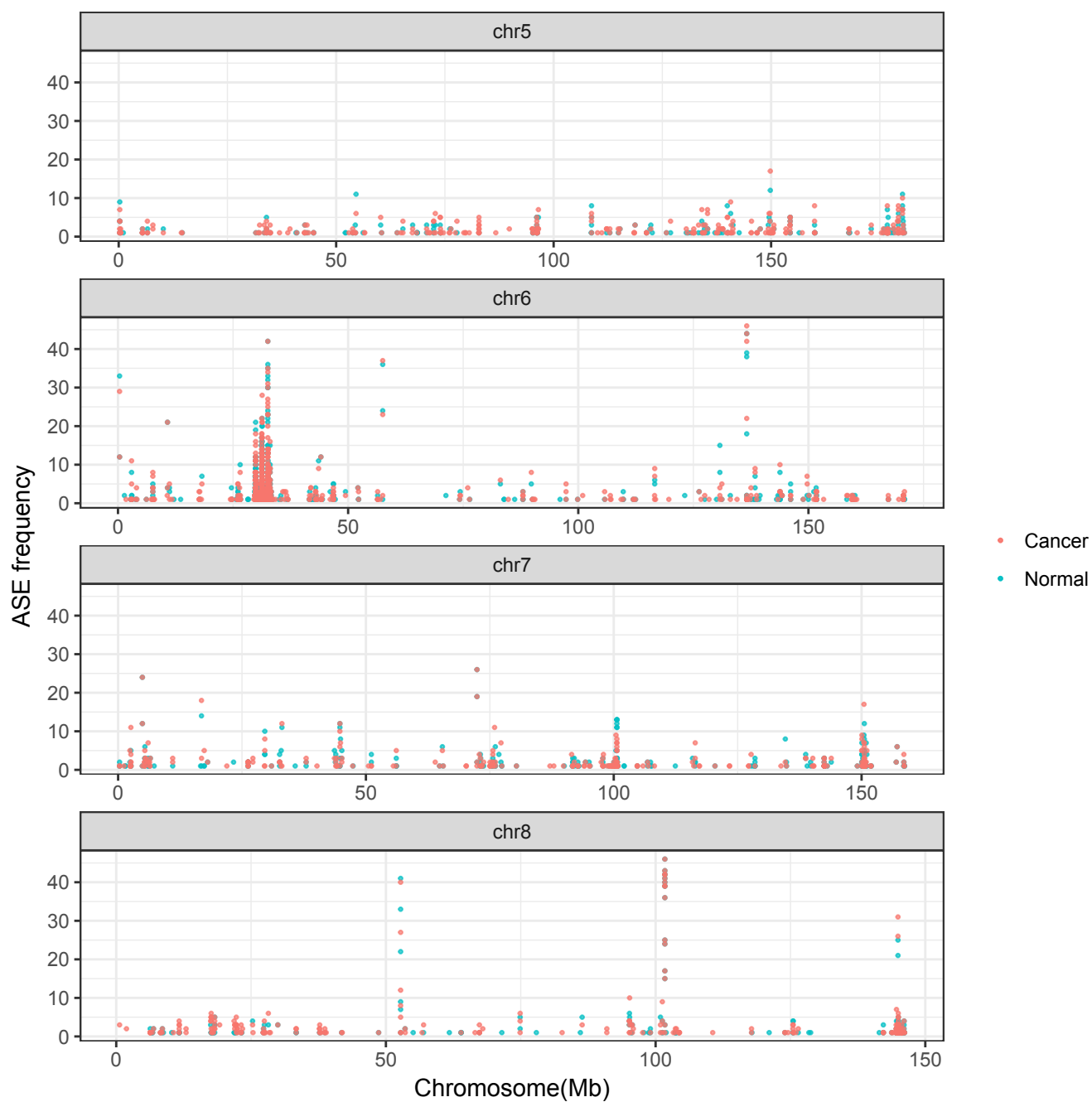

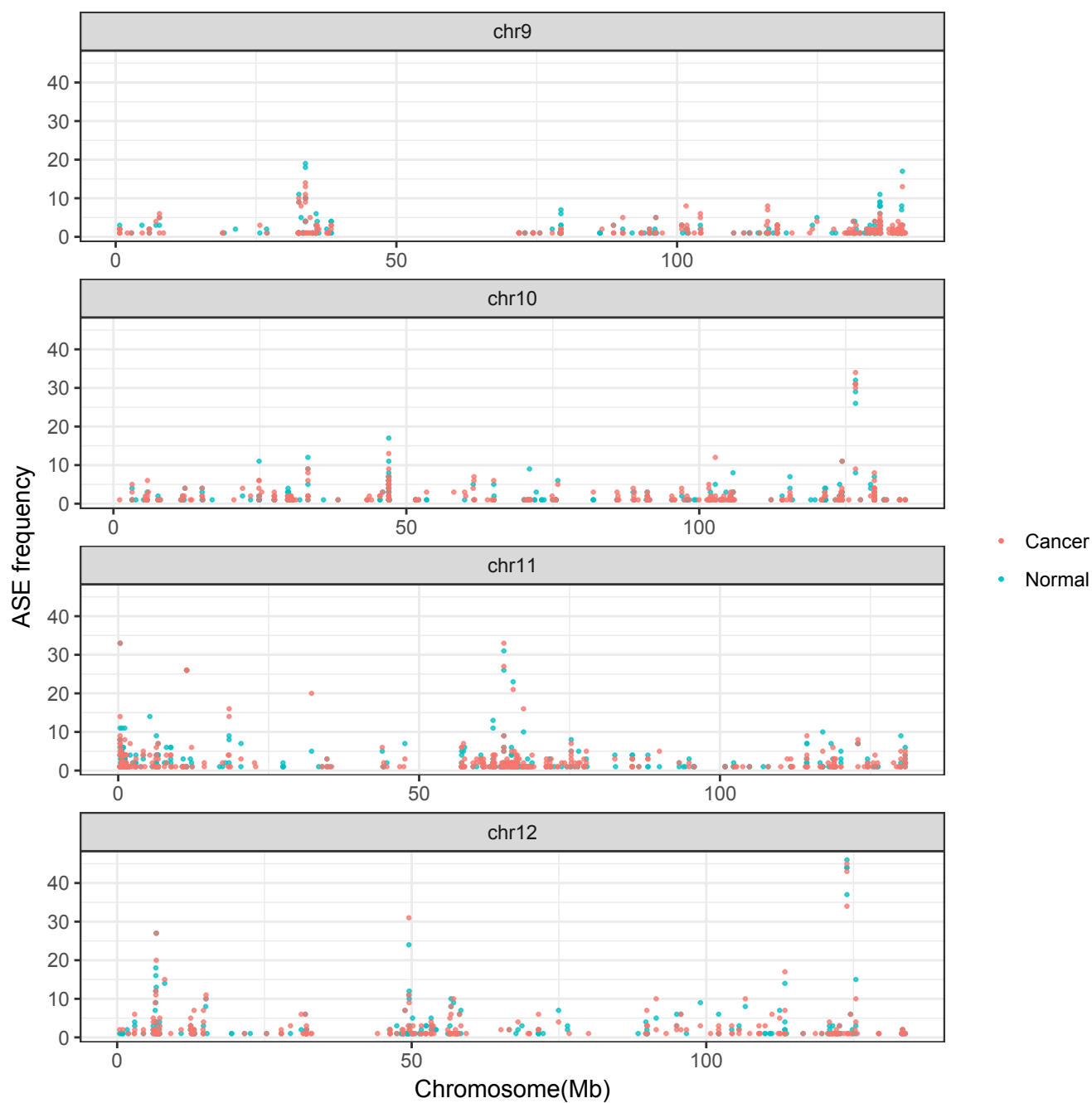

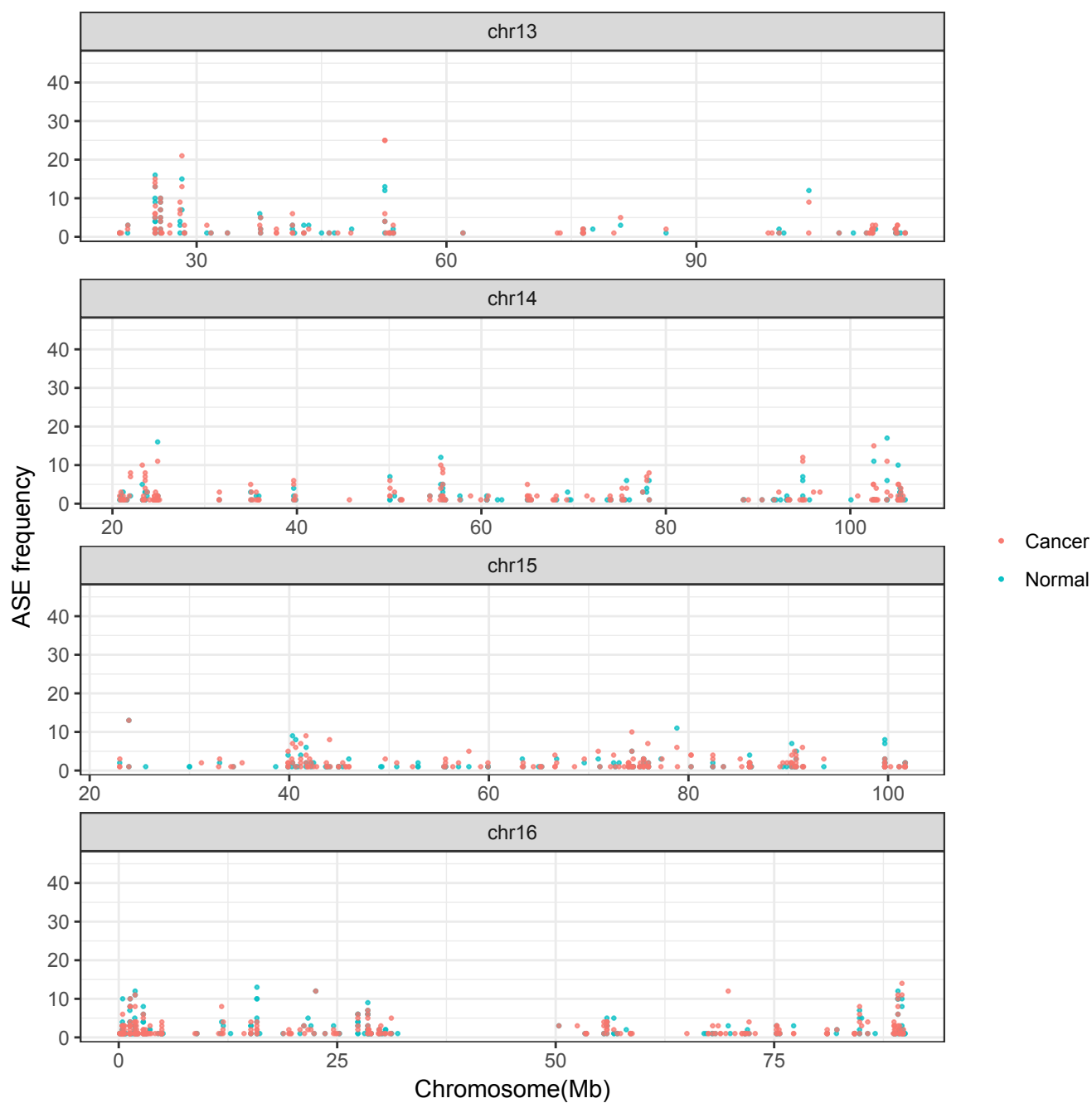

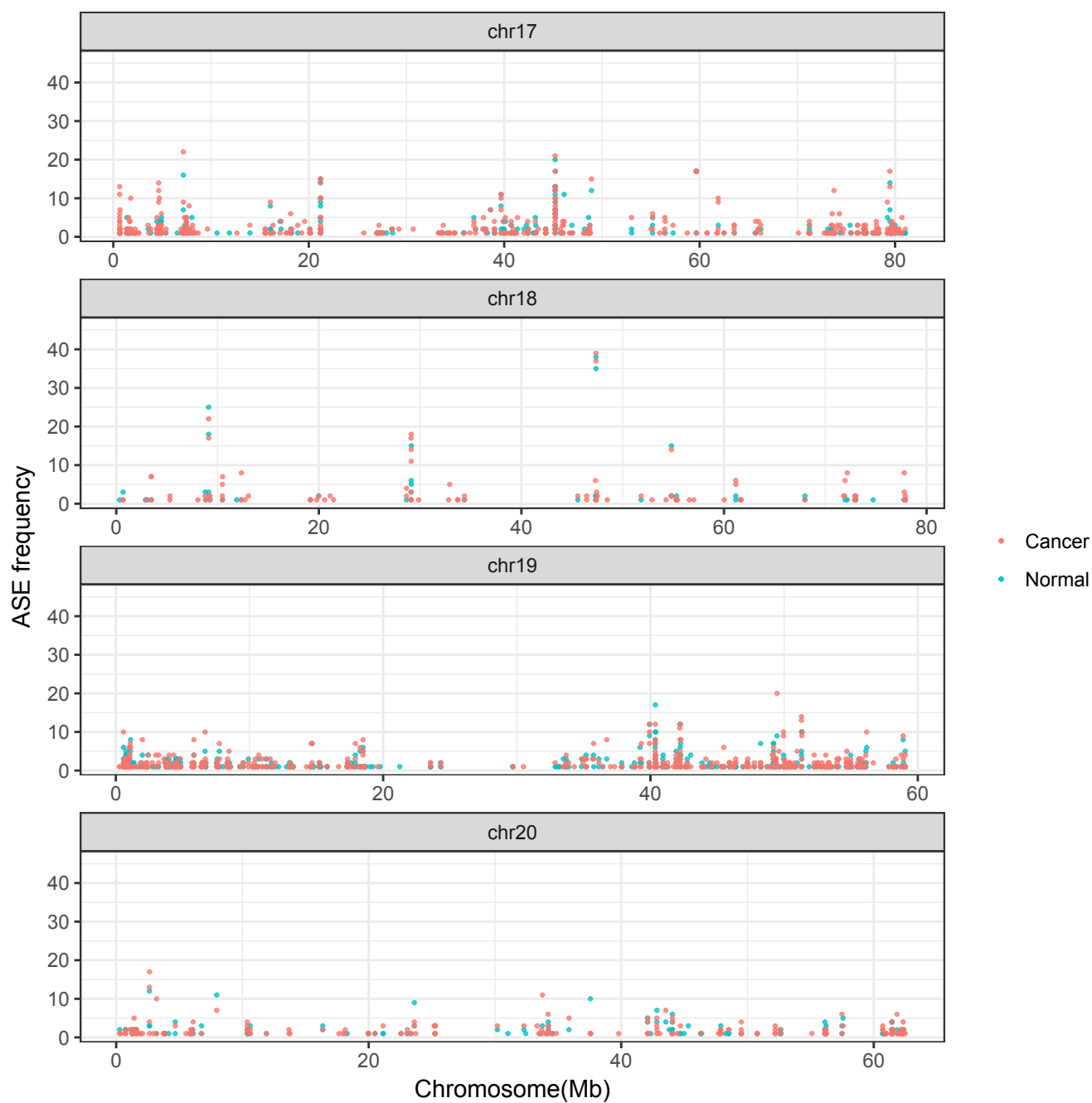

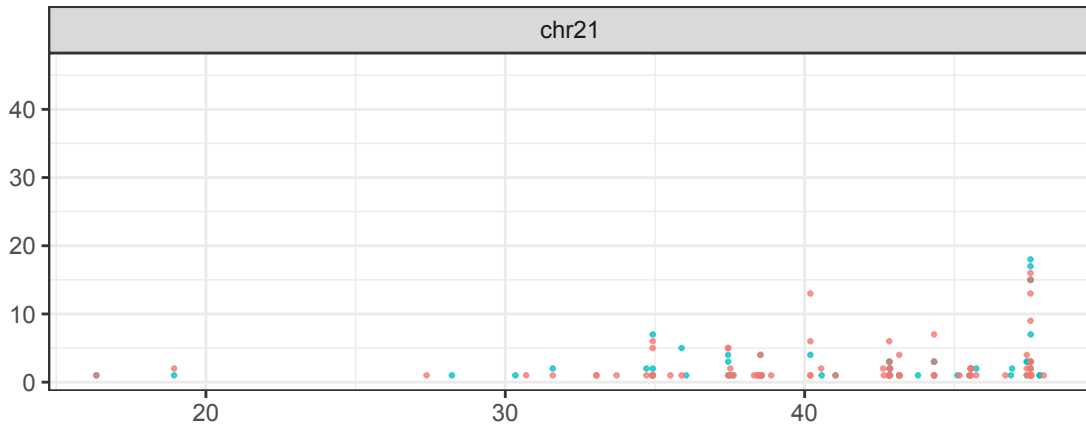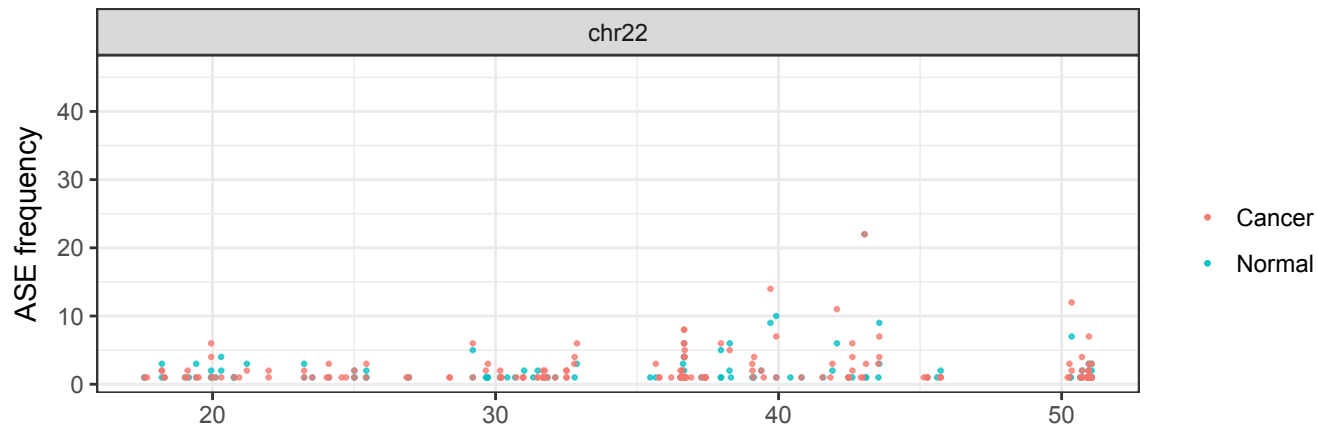

Supplement: FIGURE S2 — ASE frequency across chromosomes. [file Data_Sheet_2.PDF]

Major allele frequency

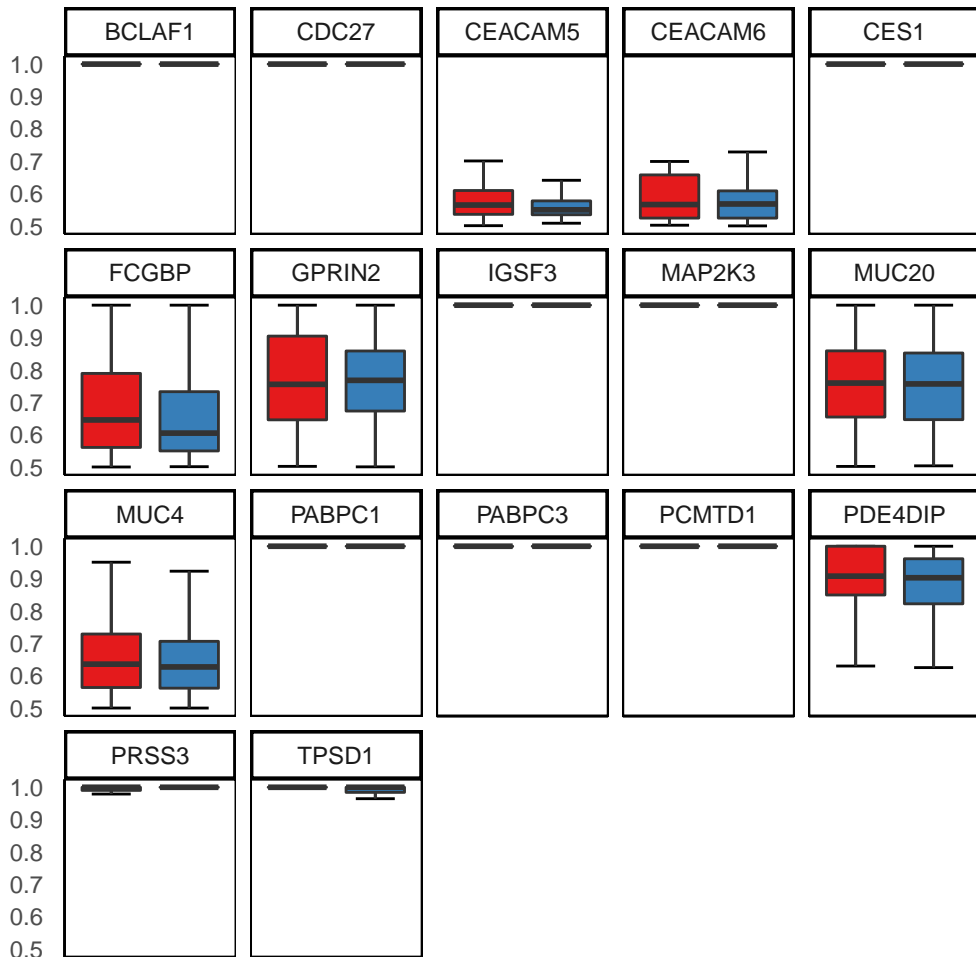

Tumor Normal

Supplement: FIGURE S3 — The comparison of allele ration of 18 shared ASE genes between normal and tumor tissues. [file Data_Sheet_3.PDF]
